# Low body mass index adversely affects the live birth rate following fresh embryo transfer in Chinese women with polycystic ovary syndrome: a pilot study

**DOI:** 10.3389/fendo.2025.1624389

**Published:** 2025-08-29

**Authors:** Nianjun Su, Lijing Wang, Ruiqiong Zhou, Yang Liao, Juan Huang, Cuiyu Huang, Yangcheng Yao, Quan Qi, Li Huang, Xiqian Zhang, Fenghua Liu

**Affiliations:** ^1^ Department of Reproductive Health and Infertility, Guangdong Province Women and Children Hospital, Guangzhou, China; ^2^ Department of Reproductive Health and Infertility, Zhaoqing Maternal and Child Health Care Hospital, Zhaoqing, China; ^3^ Guangzhou Medical University, Guangzhou, China

**Keywords:** body mass index, polycystic ovary syndrome, fresh embryo transfer, live birth rate, fitting curve

## Abstract

**Objective:**

To examine the relationship between body mass index (BMI) and pregnancy outcomes after fresh embryo transfer in patients with polycystic ovary syndrome (PCOS).

**Methods:**

Women diagnosed with PCOS who underwent *in vitro* fertilization or intracytoplasmic sperm injection treatment at the Reproductive Center of Guangdong Maternal and Child Health Hospital in China between August 2014 and July 2023 were included. Patients were divided into four groups based on BMI: Low <18.5Kg/m2 (group 1); Normal 18.5≤BMI <24 Kg/m2(group 2); Overweight 24≤BMI <28 Kg/m2(group 3); Obesity ≥28 Kg/m2(group 4). The retrospective cohort study explored the relationship between BMI and pregnancy outcomes using a logistic regression approach. The live birth rates of different BMI groups were compared after adjusting for age, antimullerian hormone (AMH), antral follicle count (AFC), homeostatic model assessment of insulin resistance (HOMA), estrogen (E2), total testosterone (T), baseline follicle-stimulating hormone (bFSH), and baseline luteinizing hormone (bLH).

**Results:**

A total of 649 patients with PCOS who underwent fresh embryo transfer were included. A curvilinear relationship was observed between BMI and pregnancy outcomes. Compared with the normal BMI group, the low BMI group had the lowest live birth rate (*P* = 0.021). An inflection point was identified at approximately 22 kg/m². When BMI was below 22 kg/m², the live birth rate increased by 29% for each 1 kg/m² increase in BMI (*P* = 0.018). When BMI exceeded 22 kg/m², the live birth rate plateaued. A significant interaction between age and BMI was also observed in relation to live birth rate (*P* = 0.011), with the adverse effect of low BMI being particularly pronounced in younger patients.

**Conclusion:**

Among patients with PCOS, those with low BMI had the lowest live birth rate following fresh embryo transfer. The optimal BMI associated with the highest live birth rate was approximately 22 kg/m².

## Introduction

Polycystic ovary syndrome (PCOS) is a common cause of female infertility, with a global prevalence ranging from 4% to 21% (1). In China, the prevalence increased from 5.6% in 2013 to 7.8% in 2022 (2, 3). PCOS is more prevalent among women of reproductive age. Patients with PCOS constitute a substantial proportion of those undergoing assisted reproductive technology (ART), and their endocrine and metabolic abnormalities may influence ART outcomes. PCOS is a metabolic disorder characterized by endocrine abnormalities. Compared to age-matched individuals without PCOS, those with PCOS are more likely to be overweight or obese (4). Previous studies have shown that, although the clinical pregnancy rate in obese women undergoing *in vitro* fertilization (IVF) is similar to that in women of normal weight, women with both PCOS and obesity have a relatively higher miscarriage rate (5, 6), which contributes to a reduced live birth rate (7). In recent years, increasing attention has been paid to the impact of body mass index (BMI) on assisted reproduction outcomes. However, BMI classification is inconsistent across studies, and most investigations examining the impact of BMI on pregnancy outcomes in patients with PCOS are based on the World Health Organization (WHO) criteria, which are primarily derived from European populations. Given that the standard BMI cut-off may underestimate obesity-related risks in Asian populations, the WHO redefined the classification for the Asia–Pacific region in 2000: overweight as BMI ≥ 23 kg/m² and obesity as BMI ≥ 25 kg/m² (8). Previous studies have also shown that the prevalence of metabolic abnormalities increases significantly in individuals with a BMI ≥ 23 kg/m², supporting this threshold as a valid cut-off for identifying those at high risk of metabolic complications, including Chinese women with PCOS (9, 10). However, the Chinese Obesity Working Group classifies BMI as follows: underweight, < 18.5 kg/m²; normal, 18.5 ≤ BMI < 24 kg/m²; overweight, 24 ≤ BMI < 28 kg/m²; and obesity, ≥ 28 kg/m² (11). Liang H et al. also demonstrated that this stratification system is appropriate for the Chinese PCOS population (12). Given the potential for BMI abnormalities in many patients with PCOS, further investigation is needed to establish a more accurate BMI cut-off point for optimizing assisted reproduction outcomes in different populations.

Women with PCOS have high rates of obesity, and most exhibit abdominal obesity, which is more likely to result in metabolic abnormalities, particularly insulin resistance (13). These metabolic disturbances affect not only overweight or obese PCOS patients but also those with normal weight (14). An WHO survey reported that Asians tend to have lower BMI but higher body fat percentages, along with increased risks of diabetes and cardiovascular disease (15). A study conducted by the Obstetrics and Gynecology Hospital of Fudan University in China also demonstrated that some non-obese women with PCOS exhibited high body fat and low muscle mass. Although their BMI was within or below the normal range, they still presented with insulin resistance (16). Previous studies have emphasized the adverse effects of obesity on IVF outcomes, including reduced live birth rates and increased risk of miscarriage (17). However, there remains a significant lack of research on assisted reproductive outcomes specifically in non-obese patients with PCOS.

Generally, if patients with PCOS present with excessively high BMI values, most ART centers recommend BMI adjustment before initiating treatment, which may lead to delays in conception and increased patient anxiety. Therefore, identifying an optimal BMI cut-off is essential for guiding clinical decision-making. In this study, we compared the pregnancy outcomes of PCOS patients with varying BMI levels following IVF–ET, including clinical pregnancy rate, early miscarriage rate, ongoing pregnancy rate, and live birth rate, in order to determine the most appropriate BMI cut-off—indicating the target BMI value during the preconception management phase.

## Materials and methods

### Subjects

This study was a retrospective cohort study approved by the Institutional Review Committee of Guangdong Maternal and Child Health Hospital. It included data from patients with PCOS who underwent *in vitro* fertilization or intracytoplasmic sperm injection (IVF/ICSI) and controlled ovarian hyperstimulation using an antagonist regimen at the Reproductive Center of Guangdong Maternal and Child Health Hospital between August 2014 and July 2023. All participants provided written informed consent. This study adhered to relevant guidelines and regulations and complied with the Guidelines for Strengthening the Reporting of Observational Studies in Epidemiology. Inclusion criteria were: (1) diagnosis of polycystic ovary syndrome; and (2) treatment with IVF/ICSI. Exclusion criteria were: history of ovarian surgery, age over 40 years, recurrent miscarriage, congenital or acquired reproductive tract malformations, uncontrolled hypertension, diabetes, thyroid disorders, or other significant medical conditions. Patients who did not undergo fresh embryo transfer for any reason were also excluded.

All data were retrieved from an independent electronic medical record system at the Reproductive Center. A database search was conducted using the terms “PCOS” and “IVF/ICSI,” and results were manually reviewed to ensure completeness and accuracy.

### Treatment plan

All patients underwent controlled ovarian hyperstimulation using antagonist protocols. Beginning on days 2 to 4 of the menstrual cycle, gonadotropins (75–250 IU/day) were administered. The initial gonadotropin dose was determined based on patient age, ovarian reserve markers—including antral follicle count (AFC), basal follicle-stimulating hormone (bFSH), anti-Müllerian hormone (AMH)—BMI, and prior ovarian stimulation history. After 4–5 days of stimulation, the type and dose of gonadotropins were adjusted according to follicular development. Two antagonist protocols were used. In the flexible protocol, a gonadotropin-releasing hormone antagonist (0.25 mg/day) was initiated when the leading follicle reached 12 mm in diameter and continued until the day of human chorionic gonadotropin (HCG) administration. In the fixed protocol, the same dose of antagonist was initiated on day 5 of stimulation and continued to the HCG day. If the serum luteinizing hormone (LH) level was below 1.0 IU/mL, supplemental high-purity urinary FSH, human menopausal gonadotropin or recombinant luteinizing hormone could be administered if necessary.

When at least two follicles reached a diameter of ≥18 mm or three follicles reached ≥17 mm, based on transvaginal ultrasound and serum hormone levels (estradiol [E2], LH, and progesterone [P]), final oocyte maturation was triggered. Triggering agents were selected according to the patient’s risk of ovarian hyperstimulation syndrome: either a single intramuscular injection of HCG (5,000–10,000 IU) or a dual trigger using triptorelin (0.2 mg) combined with HCG (1,000–2,000 IU). Oocyte retrieval was performed 36 hours later. Fertilization was performed via IVF, ICSI, or both. Before July 2017, a self-prepared culture medium was used, which was subsequently replaced by Kato culture medium. According to internal quality control, no significant differences in clinical outcomes were observed before and after the media change.

After oocyte retrieval, the decision to proceed with a fresh embryo transfer was based on several factors, including fertilization status, serum E2 levels, presence of ascites, endometrial condition, and overall patient status. If conditions were deemed suitable, no more than two cleavage-stage embryos or one blastocyst was transferred. Embryo quality was assessed using a standardized protocol that monitored the morphology of oocytes and early embryos during *in vitro* culture. Cleavage-stage embryos with at least five blastomeres were considered transferable, and those with six to ten blastomeres were classified as high-quality embryos. Blastocysts were evaluated according to Gardner’s criteria (18); those graded 3BB or higher were regarded as high-quality blastocysts.

### Pregnancy outcomes

We applied the 2017 consensus definitions established by the American Society for Reproductive Medicine to classify clinical outcomes (19). Clinical pregnancy was defined as a pregnancy confirmed by the presence of one or more gestational sacs or other definitive signs on ultrasound. Early miscarriage was defined as pregnancy loss before 12 weeks of gestation following ultrasound confirmation of an intrauterine gestational sac. Ongoing pregnancy was defined as pregnancy continuing beyond 12 weeks of gestation. A live birth was defined as the delivery of at least one live infant.

### Covariates

The selection of potential confounders was based on previous literature (10, 12) and supplemented by covariates found to be significant in the univariate analysis (**Table 1**). The final adjusted model included the following variables: age, AMH, AFC, homeostatic model assessment of insulin resistance (HOMA), bFSH, bLH, LH/FSH ratio, E2, and testosterone (T).

**Table 1:** Univariate analysis.

Variable	OR_95%CI	P_value
Age (y)	0.99 (0.95~1.03)	0.618
Wt (Kg)	1.00 (0.98~1.02)	0.947
Ht (cm)	1.01 (0.97~1.04)	0.680
WC (cm)	0.99 (0.97~1.01)	0.370
HC (cm)	0.98 (0.96~1.01)	0.262
WHR	1.35 (0.05~33.8)	0.857
AMH (ng/ml)	0.99 (0.96~1.02)	0.473
AFC	0.99 (0.97~1.02)	0.448
bFSH (IU/L)	1.12 (1.01~1.24)	**0.038**
bLH (IU/L)	0.97 (0.94~1.00)	**0.061**
bLH/bFSH	0.78 (0.63~0.95)	**0.014**
bE2 (pg/ml)	0.99 (0.99~1.00)	**0.086**
bP4 (ng/ml)	1.01 (0.91~1.12)	0.833
bPRL (ng/ml)	0.99 (0.98~1.01)	0.316
bT (ng/ml)	0.37 (0.14~0.96)	**0.041**
AND (nmol/L)	0.98 (0.93~1.02)	0.243
HOMA	1.02 (0.91~1.14)	0.773

Wt, weight; Ht, height; WC, waist circumference; HC, hip circumference; WHR, Waist-to-Hip Ratio; AMH, Antimullerian hormone; AFC antral follicle count; b.FSH, baseline follicle stimulating hormone; b.LH, baseline luteinizing hormone; b.E2, baseline estradiol; b.T, baseline testosterone; b.PRL, baseline prolactin; b.P4, progesterone; AND, androstenedione; HOMA, Homeostatic Model Assessment of Insulin Resistance.

Bold font is used to denote statistically significant values (defined as p < 0.1).

### Statistical analysis

Continuous variables with a normal distribution were presented as mean ± standard deviation, while those with non-normal distributions were expressed as median (25th percentile, 75th percentile). Categorical variables were described using proportions. Univariate comparisons between groups were conducted using the Kruskal–Wallis H test for continuous variables and the Pearson chi-square test for categorical variables.

Logistic regression was used to evaluate the association between BMI and the clinical pregnancy rate, early miscarriage rate, ongoing pregnancy rate, and live birth rate. Fitting curves and nonlinear tests were employed to assess the relationship between BMI and live birth rate, and stratified analyses were conducted to explore age-specific associations. A two-sided p-value < 0.05 was considered statistically significant. Statistical analyses were performed using R statistical software (version 4.2.2; R Foundation for Statistical Computing, Vienna, Austria; http://www.R-project.org) and the Free Statistics Analysis Platform (version 2.1; Beijing, China; http://www.clinicalscientists.cn/freestatistics).

## Results

### Baseline characteristics of participants

**Figure 1:**
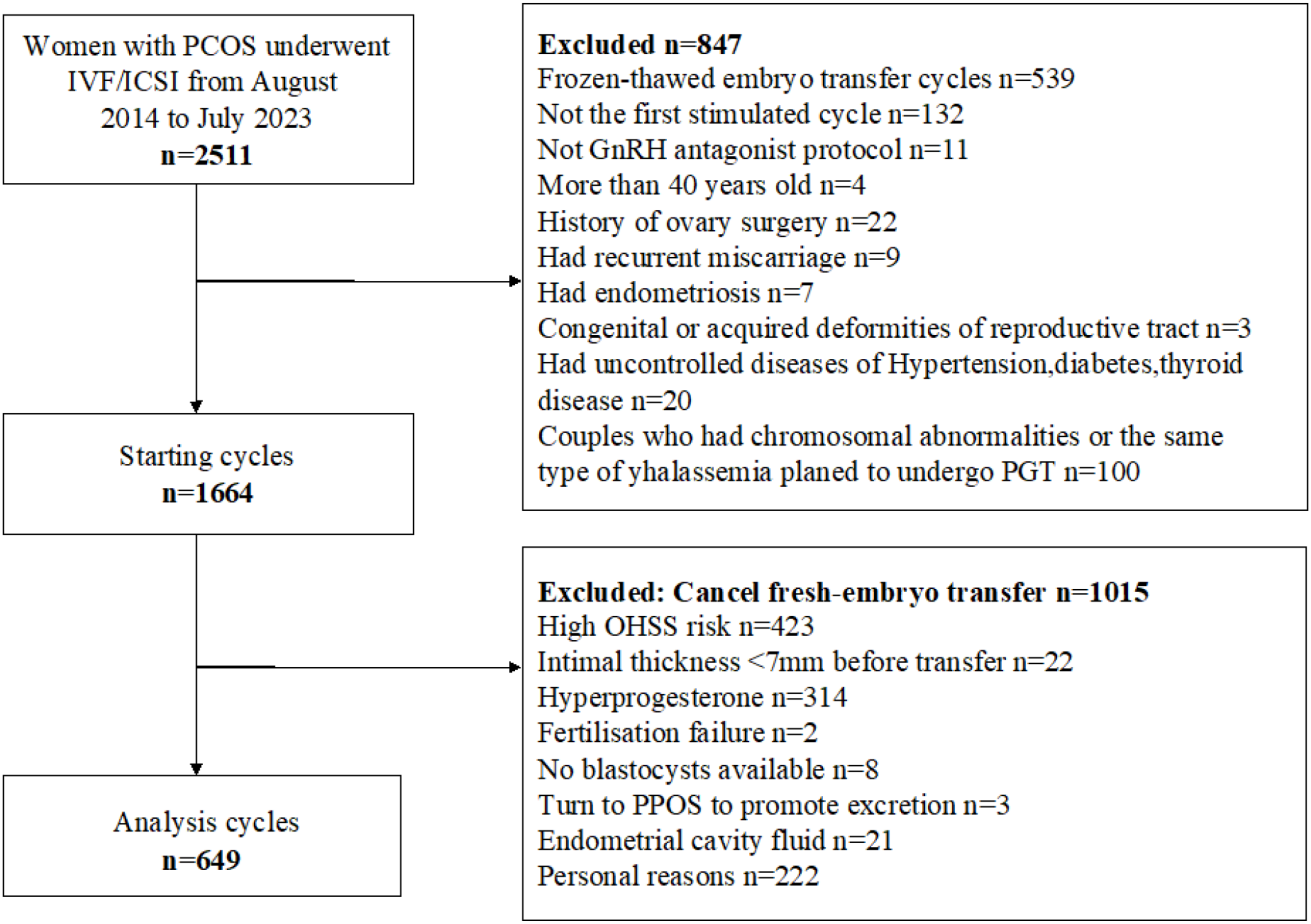
Flow Chart of the study cohort.

As shown in **Figure 1**, 649 patients who met the inclusion criteria were analyzed. **Table 2** presents the demographic characteristics of the study population. Among women with PCOS, 6.8% had low BMI, 57.9% had normal BMI, 25.7% were overweight, and 9.6% were obese. Significant differences were observed among BMI groups in waist circumference, waist-to-hip ratio, body weight, and homeostasis model assessment of insulin resistance (P < 0.05). Differences were also noted in ovarian function–related indicators, including AMH, bFSH, basal luteinizing hormone (bLH), basal estradiol (bE2), and basal progesterone (bP4) levels (P < 0.05). Additionally, ovarian superovulation–related variables, such as the initiation dose of gonadotropin, the total gonadotropin dose per cycle, and the number of oocytes retrieved, varied significantly between BMI subgroups (P < 0.05). However, no significant differences were found in height, AFC, prolactin, or testosterone levels among the BMI categories.

**Table 2:** Baseline characteristics of the study population.

Variables	BMI					P value
	Total (n = 649)	<18.5 (n = 44)	18.5-23.9 (n = 376)	24-27.9 (n = 167)	≥28 (n = 62)	
Age (y)	29.7 ± 3.6	28.0 ± 2.9	29.5 ± 3.5	30.2 ± 3.7	30.5 ± 4.1	**< 0.001**
Wt (Kg)	57.7 ± 9.1	44.3 ± 3.5	53.8 ± 4.6	63.5 ± 4.4	75.5 ± 6.9	**< 0.001**
Ht (cm)	158.2 ± 4.6	159.2 ± 5.1	158.2 ± 4.4	158.0 ± 4.6	157.9 ± 5.6	0.502
WC (cm)	78.1 ± 8.6	67.4 ± 5.3	75.2 ± 6.1	82.3 ± 6.5	92.7 ± 5.8	**< 0.001**
HC (cm)	92.7 ± 7.1	85.2 ± 5.5	90.0 ± 4.8	96.4 ± 5.0	104.9 ± 5.9	**< 0.001**
WHR	0.84 ± 0.06	0.79 ± 0.04	0.84 ± 0.06	0.85 ± 0.05	0.89 ± 0.06	**< 0.001**
AMH (ng/ml)	9.3 ± 4.7	10.7 ± 5.0	9.6 ± 4.6	9.2 ± 5.0	6.7 ± 3.5	**< 0.001**
AFC	24.7 ± 6.3	24.6 ± 5.6	24.5 ± 6.5	25.1 ± 6.5	25.2 ± 5.3	0.731
bFSH (IU/L)	6.3 ± 1.5	6.9 ± 1.5	6.3 ± 1.5	6.1 ± 1.5	5.8 ± 1.3	**0.001**
bLH (IU/L)	8.7 ± 4.8	10.9 ± 5.9	9.1 ± 4.8	8.1 ± 4.1	7.0 ± 4.3	**< 0.001**
bLH/bFSH	1.4 ± 0.8	1.6 ± 0.9	1.5 ± 0.9	1.3 ± 0.6	1.2 ± 0.7	**0.012**
bE2 (pg/ml)	41.0 ± 26.1	53.1 ± 41.6	41.1 ± 26.0	39.7 ± 22.6	35.3 ± 17.5	**0.005**
bP4 (ng/ml)	0.28 (0.14, 0.47)	0.38 (0.24, 0.58)	0.27 (0.14, 0.46)	0.30 (0.15, 0.49)	0.20 (0.08, 0.43)	**0.004**
bE2/bP4	219.3 ± 241.4	202.5 ± 221.5	219.7 ± 245.5	202.2 ± 235.1	272.3 ± 243.8	0.256
bPRL (ng/ml)	18.8 ± 10.2	21.2 ± 9.2	18.7 ± 9.8	18.6 ± 10.4	17.7 ± 12.2	0.354
bT (ng/ml)	0.37± 0.43	0.32 ± 0.14	0.37 ± 0.54	0.40 ± 0.19	0.37 ± 0.18	0.694
AND (nmol/L)	11.4 ± 5.2	11.3 ± 3.4	11.4 ± 5.6	11.9 ± 5.0	9.9 ± 4.0	0.315
HOMA	2.22 (1.59, 3.18)	1.39 (1.24, 1.86)	1.94 (1.48, 2.73)	2.72 (2.19, 3.82)	4.14 (2.92, 5.11)	**< 0.001**
Gn_TolDose (IU)	1488.0 ± 581.7	1161.7 ± 418.1	1370.2 ± 507.0	1639.1 ± 556.1	2013.7 ± 728.2	**< 0.001**
Gn_TolDose/BMI	64.5 ± 22.7	67.1 ± 26.3	63.8 ± 22.6	64.5 ± 22.0	66.9 ± 22.6	0.644
Start_Dose (IU)	125.0 ± 28.1	110.1 ± 20.5	120.3 ± 25.1	130.8 ± 27.9	148.2 ± 33.4	**< 0.001**
Start_Dose/BMI	5.5 ± 1.2	6.3 ± 1.4	5.6 ± 1.2	5.2 ± 1.1	4.9 ± 1.1	**< 0.001**
NOR	13.0 ± 5.3	13.7 ± 4.1	13.4 ± 5.4	12.4 ± 5.5	11.1 ± 5.2	**0.005**
Fertilization methods						0.140
IVF	76.6 (497/649)	79.5 (35/44)	75.5 (284/376)	80.8 (135/167)	69.4 (43/62)	
ICSI	23.1 (150/649)	20.5 (9/44)	24.5 (92/376)	18.6 (31/167)	29 (18/62)	
IVF+ICSI	0.3 (2/649)	0 (0/44)	0 (0/376)	0.6 (1/167)	1.6 (1/62)	

Data are presented as mean ± standard deviation (SD) for continuous variables with normal distribution or as median (IQR) for continuous variables that did not show a normal distribution, and categorical variables are reported as no. (%). BMI, Body Mass Index; Wt, weight; Ht, height; WC, waist circumference; HC, hip circumference; WHR, Waist-to-Hip Ratio; AMH, Antimullerian hormone; AFC antral follicle count; b.FSH, baseline follicle stimulating hormone; b.LH, baseline luteinizing hormone; b.E2, baseline estradiol; b.T, baseline testosterone; b.PRL, baseline prolactin; b.P4, progesterone; AND, androstenedione; HOMA, Homeostatic Model Assessment of Insulin Resistance; Gn_TolDose, gonadotropin total dose;Start_Dose,gonadotropin start dose; NOR, number of retrieved oocytes; IVF, *in vitro* fertilization; ICSI, intracytoplasmic sperm injection.

Bold font is used to denote statistically significant values (defined as p < 0.05).

### BMI and pregnancy outcomes

As shown in **Table 3**, multivariate logistic regression models demonstrated associations between BMI and pregnancy outcomes. Based on previous literature and univariate analysis, four models (Model 1–4) were constructed. In Model 1, compared with the normal BMI group, the low BMI group showed a significantly lower live birth rate (P = 0.021; odds ratio [OR], 0.47; 95% confidence interval [CI], 0.24–0.89), a significantly lower clinical pregnancy rate (P = 0.025; OR, 0.49; 95% CI, 0.26–0.91), and a significantly lower ongoing pregnancy rate (P = 0.008; OR, 0.41; 95% CI, 0.22–0.79). In Model 4, after adjusting for confounding variables including age, AMH, AFC, HOMA, and baseline endocrine indicators (bFSH, bLH, bLH/bFSH ratio, bE2, and testosterone [bT]), the early miscarriage rate in the low BMI group was significantly higher than that in the normal BMI group (P = 0.040; OR, 4.33; 95% CI, 1.07–17.50). Compared with women with normal BMI (55.1%), the live birth rate in the obese group (46.8%) was lower, although the difference was not statistically significant.

**Table 3:** Logistic multivariate analysis of pregnancy outcomes and Body Mass Index.

Variable	n.event_%	Model 1 OR(95%CI)	P	Model 2 OR(95%CI)	P	Model 3 OR(95%CI)	P	Model 4 OR(95%CI)	P
Clinical pregnancy rate									
BMI<18.5	22 (50.0)	0.49 (0.26~0.91)	**0.025**	0.48 (0.25~0.90)	**0.021**	0.49 (0.25~0.96)	**0.039**	0.48 (0.24~0.97)	**0.042**
18.5≤BMI<24	253 (67.3)	1(Ref)		1(Ref)		1(Ref)		1(Ref)	
24≤BMI<28	113 (67.7)	1.02 (0.69~1.50)	0.931	1.02 (0.69~1.50)	0.935	1.15 (0.74~1.80)	0.537	1.18 (0.74~1.88)	0.484
BMI≥28	37 (59.7)	0.72 (0.41~1.25)	0.242	0.76 (0.44~1.31)	0.324	0.75 (0.38~1.49)	0.409	0.81 (0.40~1.62)	0.550
Early miscarriage rate									
BMI<18.5	4 (9.1)	2.67 (0.82~8.70)	0.103	2.98 (0.90~9.84)	0.074	3.97 (1.08~14.63)	**0.038**	4.33 (1.07~17.50)	**0.040**
18.5≤BMI<24	20 (5.3)	1(Ref)	20 (5.3)	1(Ref)		1(Ref)		1(Ref)	
24≤BMI<28	8 (4.8)	0.88 (0.38~2.07)	0.772	0.86 (0.36~2.02)	0.725	0.70 (0.25~2.00)	0.508	0.72 (0.24~2.13)	0.555
BMI≥28	3 (4.8)	1.10 (0.31~3.91)	0.885	0.98 (0.27~3.51)	0.974	0.80 (0.14~4.67)	0.801	0.78 (0.13~4.62)	0.786
On-going pregnancy rate									
BMI<18.5	16 (36.4)	0.41 (0.22~0.79)	**0.008**	0.39 (0.20~0.75)	**0.005**	0.37 (0.19~0.76)	**0.006**	0.34 (0.16~0.70)	**0.004**
18.5≤BMI<24	218 (58)	1(Ref)		1(Ref)		1(Ref)		1(Ref)	
24≤BMI<28	101 (60.5)	1.11 (0.76~1.61)	0.585	1.13 (0.77~1.64)	0.534	1.30 (0.85~2.00)	0.231	1.33 (0.85~2.10)	0.214
BMI≥28	30 (48.4)	0.68 (0.40~1.16)	0.16	0.74 (0.43~1.26)	0.267	0.68 (0.35~1.32)	0.252	0.73 (0.37~1.46)	0.376
Live birth rate									
BMI<18.5	16 (36.4)	0.47 (0.24~0.89)	**0.021**	0.45 (0.23~0.86)	**0.016**	0.44 (0.22~0.89)	**0.022**	0.41 (0.20~0.84)	**0.015**
18.5≤BMI<24	207 (55.1)	1(Ref)		1(Ref)		1(Ref)		1(Ref)	
24≤BMI<28	91 (54.5)	0.98 (0.68~1.41)	0.903	0.98 (0.68~1.42)	0.919	1.14 (0.75~1.74)	0.529	1.15 (0.74~1.79)	0.542
BMI≥28	29 (46.8)	0.72 (0.42~1.23)	0.227	0.77 (0.45~1.32)	0.341	0.69 (0.35~1.34)	0.272	0.74 (0.37~1.47)	0.394

Model 1: crude model.

Model 2: adjusted for age.

Model 3: adjusted for Model 2+AMH+AFC+HOMA.

Model 4: adjusted for Model 3+bFSH+bLH+bLH/bFSH+bE2+bT.

Bold font is used to denote statistically significant values (defined as p < 0.05).

### Inflection point in the relationship between BMI and live birth rate

**Figure 2:**
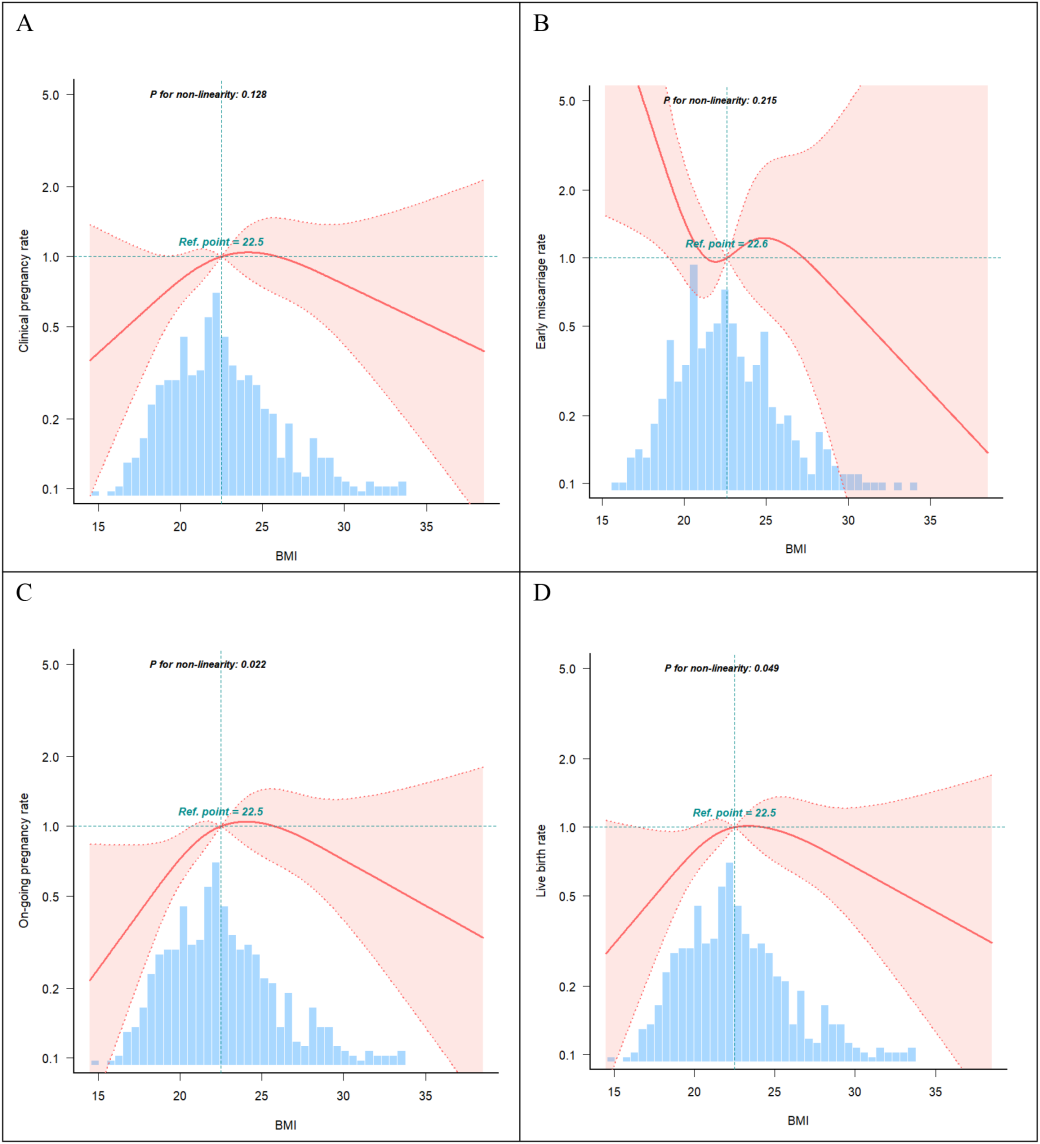
Curve fitting. **(A)** Association between BMI and clinical pregnancy rate (P >0.05). **(B)** Association between BMI and early miscarriage rate (P >0.05). **(C)** Association between BMI and on-going pregnancy rate (P <0.05). **(D)** Association between BMI and live birth rate (P <0.05). The shading portion of the image indicated the 95% confidence intervals. The odds ratio of the horizontal dashed lines were 1.0 (reference point). The reference point were the median BMI (22.5 Kg/m2). Models were adjusted for age, AMH, AFC, HOMA, bFSH, bLH, bLH/bFSH, bE2, bT.

Curve fitting revealed a non-linear relationship between BMI and live birth rate (**Figure 2**), with an inflection point at approximately 22 kg/m² (**Table 4**). To the left of the inflection point, live birth rate increased significantly with rising BMI (P = 0.018; OR, 1.29; 95% CI, 1.05–1.60). To the right of the inflection point, BMI was not significantly associated with live birth rate (P = 0.379; OR, 0.96; 95% CI, 0.87–1.06). These findings suggest that the live birth rate was highest at a BMI of approximately 22 kg/m². When BMI was below this threshold, the live birth rate increased by 29% for each 1 kg/m² increase in BMI.

**Table 4:** Inflection point analysis.

Item	Breakpoint.OR (95%CI)	P value
D_BK1	22.0 (NA,NA)	NA_character_
slope1	1.29 (1.05~1.60)	**0.018**
slope2	0.96 (0.87~1.06)	0.379
Likelihood Ratio test	–	0.013
Non-linear Test*1	–	0.005
Non-linear Test*2	–	0.029

Inflection point analysis of non-linear relationship between BMI and live birth rate.

Adjusted: age+AMH+AFC+HOMA+bFSH+bLH+LH/FSH+bE2+bT.

Bold font is used to denote statistically significant values (defined as p < 0.05).

### Subgroup and stratified analysis

**Figure 3:**
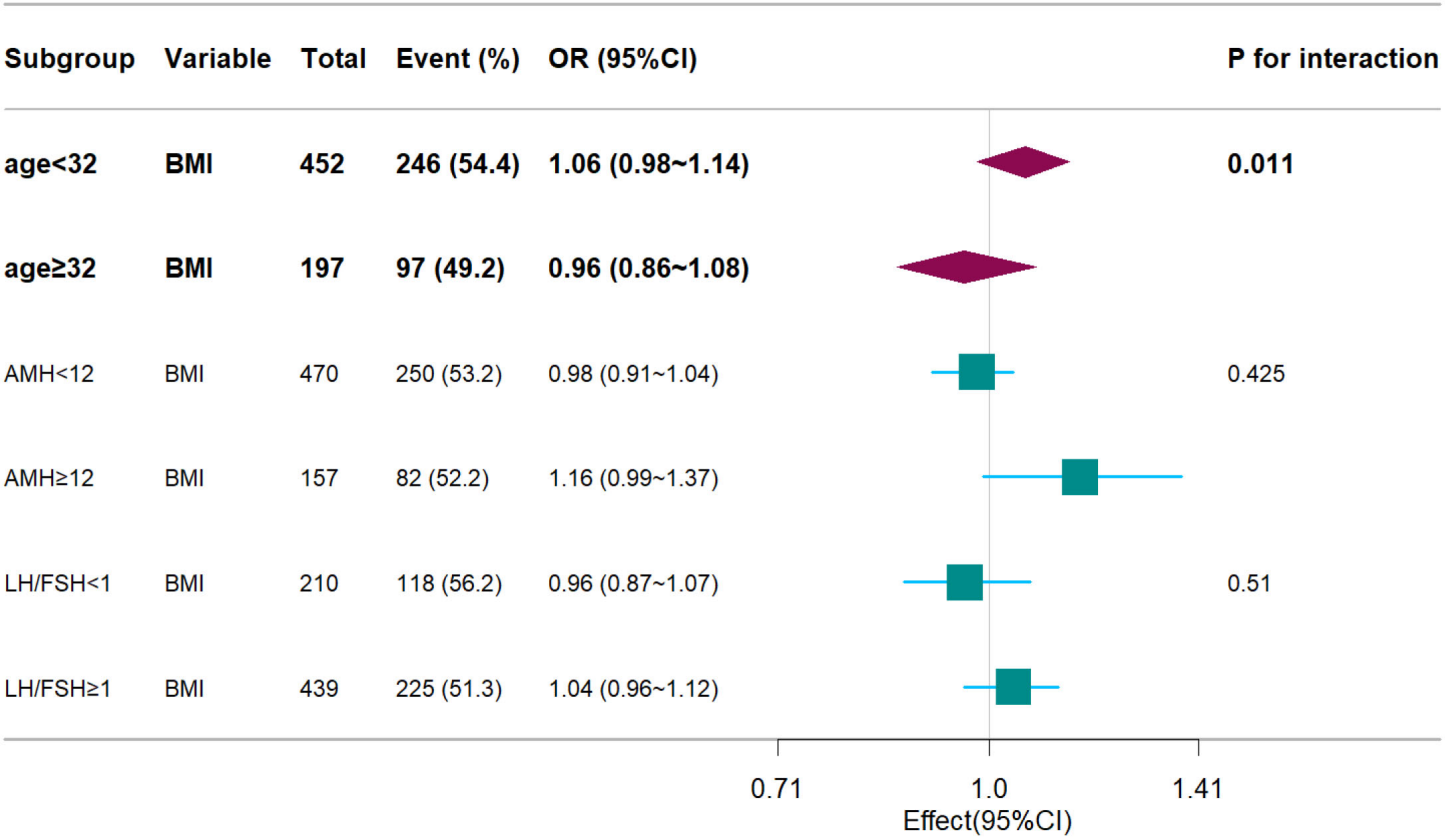
Forest plots. Associations between BMI and live birth rate, stratified by age, AMH, LH/FSH. Models were adjusted for age, AMH, AFC, HOMA, bFSH, bLH, bLH/bFSH, bE2, bT.

**Figure 4:**
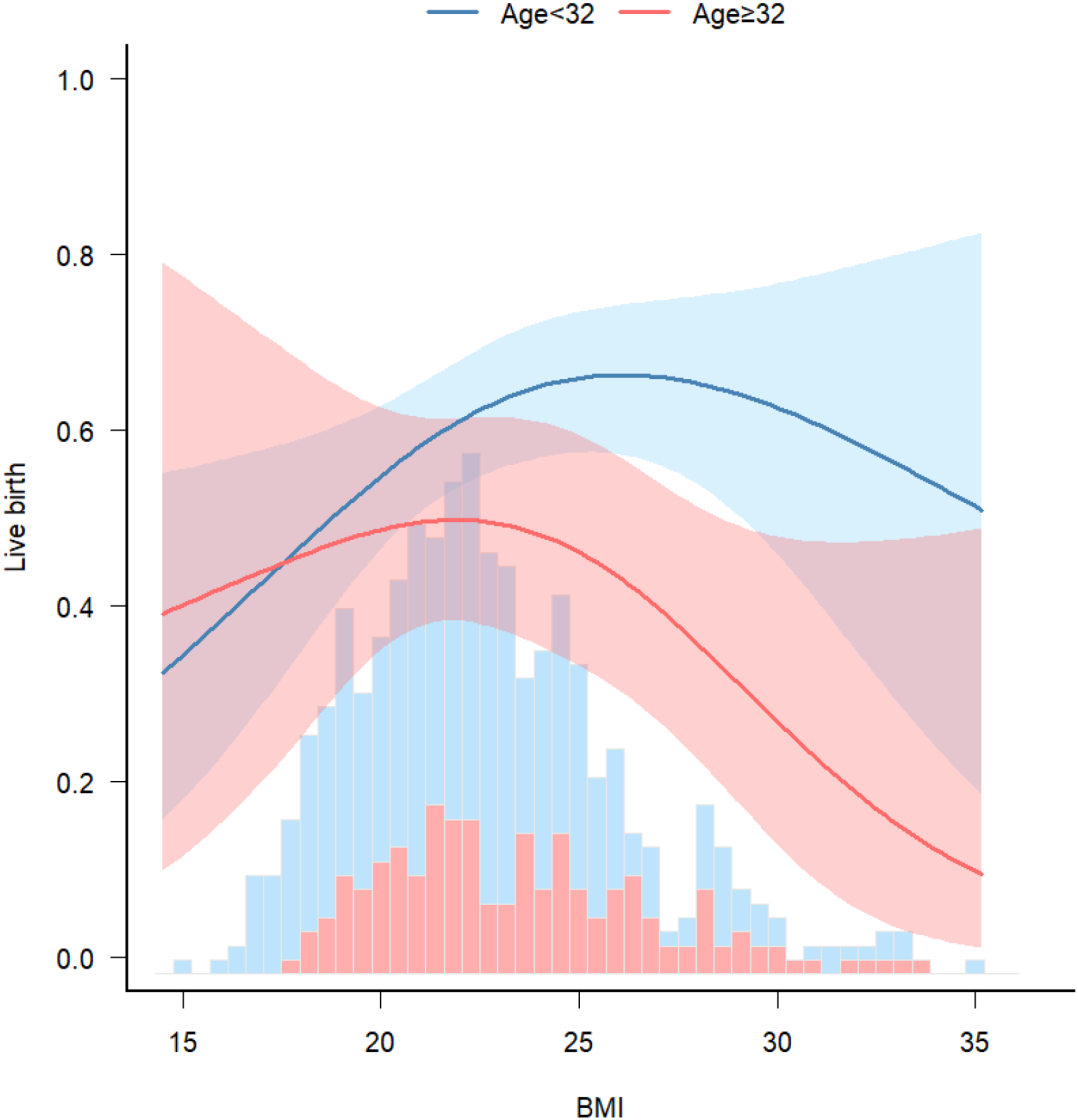
Stratified analyses. Associations between BMI and live birth rate, stratified by age. Model was adjusted for age, AMH, AFC, HOMA, bFSH, bLH, bLH/bFSH, bE2, bT.

A significant interaction was observed between age and BMI with respect to live birth rate (P = 0.011; **Table 5**, **Figures 3**, **4**). The adverse effects of low BMI on live birth rate were more pronounced in younger patients. The statistical software automatically set 32 years as the stratification point. Among patients aged < 32 years, the inflection point occurred at a BMI of 25.5 kg/m². To the left of this inflection point, live birth rate increased significantly with increasing BMI (P = 0.016; OR, 1.16; 95% CI, 1.03–1.30). To the right of the inflection point, there was no significant association between BMI and live birth rate (P = 0.581; OR, 0.96; 95% CI, 0.70–1.22; **Table 6**). Among patients aged ≥ 32 years, the inflection point was observed at a BMI of 24 kg/m². To the left of this point, no significant association was observed between BMI and live birth rate (P = 0.498; OR, 1.12; 95% CI, 0.80–1.57). However, to the right of the inflection point, live birth rate decreased significantly with increasing BMI (P = 0.020; OR, 0.71; 95% CI, 0.53–0.95; **Table 7**). The relationship between BMI and live birth rate in patients < 32 years was consistent with the trend observed in the overall population. In contrast, among patients aged ≥ 32 years, the pattern differed from the overall population trend. No significant interaction between BMI and AMH or LH/FSH was found in relation to live birth rate (**Table 5**).

**Table 5:** Subgroup analysis.

Subgroup	Variable	n.total	n.event_%	crude.OR_ 95CI	crude.P_value	adj.OR_95CI	adj.P_ value	P.for. interaction
age<32	BMI	452	246 (54.4)	1.05 (0.99~1.11)	0.110	1.06 (0.98~1.14)	0.170	**0.011**
age≥32	BMI	197	97 (49.2)	0.91 (0.84~0.99)	**0.032**	0.96 (0.86~1.08)	0.528	
AMH<12	BMI	470	250 (53.2)	1.00 (0.95~1.05)	0.984	0.98 (0.91~1.04)	0.487	0.425
AMH≥12	BMI	157	82 (52.2)	1.00 (0.89~1.12)	1.000	1.16 (0.99~1.37)	0.064	
LH/FSH<1	BMI	210	118 (56.2)	0.99 (0.92~1.07)	0.850	0.96 (0.87~1.07)	0.489	0.510
LH/FSH≥1	BMI	439	225 (51.3)	1.00 (0.94~1.05)	0.911	1.04 (0.96~1.12)	0.374	

Adjusted: age+AMH+AFC+HOMA+bFSH+bLH+LH/FSH+bE2+bT.

Bold font is used to denote statistically significant values (defined as p < 0.05).

**Table 6:** Inflection point analysis.

Item	Breakpoint.OR (95%CI)	P value
E_BK1	25.5 (NA,NA)	NA_character_
slope1	1.16 (1.03~1.30)	**0.016**
slope2	0.96 (0.70~1.22)	0.581
Likelihood Ratio test	–	0.147
Non-linear Test*1	–	0.055
Non-linear Test*2	–	0.191

Inflection point analysis of non-linear relationship between BMI and live birth rate in women aged <32 years.

Adjusted: age+AMH+AFC+HOMA+bFSH+bLH+LH/FSH+bE2+bT.

Bold font is used to denote statistically significant values (defined as p < 0.05).

**Table 7:** Inflection point analysis.

Item	Breakpoint.OR (95%CI)	P value
D_BK1	24 (NA,NA)	NA_character_
slope1	1.12 (0.80~1.57)	0.498
slope2	0.71 (0.53~0.95)	**0.020**
Likelihood Ratio test	–	0.323
Non-linear Test*1	–	0.091
Non-linear Test*2	–	0.371

Inflection point analysis of non-linear relationship between BMI and live birth rate in women aged ≥32 years.

Adjusted: age+AMH+AFC+HOMA+bFSH+bLH+LH/FSH+bE2+bT.

Bold font is used to denote statistically significant values (defined as p < 0.05).

## Discussion

In this retrospective cohort study, 649 women with PCOS underwent fresh embryo transfer following IVF/ICSI. The live birth rate was lowest in the low BMI group, and this difference was statistically significant compared with the normal BMI group. Although overweight and obese patients with PCOS did not show statistically significant differences from those with normal BMI, a trend toward lower live birth rates was observed.

Nonlinear curve analysis identified an inflection point at approximately 22 kg/m². When BMI was below this threshold, live birth rate increased progressively with increasing BMI. In contrast, when BMI exceeded 22 kg/m², the live birth rate showed a declining trend. Additionally, a significant interaction between age and BMI was observed, with the adverse effect of low BMI on live birth rate more pronounced in younger patients. These findings suggest that increasing BMI may improve pregnancy outcomes in individuals with low BMI. Conversely, in patients aged > 32 years who were overweight or obese, live birth rates were lower.

The most unexpected finding in our study was that the low BMI group exhibited both the highest miscarriage and lowest live birth rate, which contrasts with prior studies (6, 10, 12, 17). A prospective study involving 3,604 women also reported that underweight, overweight, and obese individuals had higher rates of subfertility compared with normal-weight women (20). BMI may influence IVF outcomes through several mechanisms. While healthy lifestyle modifications and weight loss are established first-line treatments for overweight or obese patients with infertility, low BMI has received comparatively less clinical attention. Notably, Asian individuals tend to have higher body fat percentages than Europeans at equivalent BMI values (21), which may lead to underestimation of the risk for metabolic complications such as diabetes and cardiovascular disease (15). A proportion of non-obese patients with PCOS exhibit high body fat, low muscle mass, abdominal obesity, and insulin resistance, despite having a BMI within the normal or even low range (16, 22). Two clinical studies have reported that lifestyle interventions—particularly those aimed at increasing muscle mass—can significantly improve insulin resistance and enhance rates of spontaneous ovulation. Several other studies have also suggested that increasing skeletal muscle mass can substantially improve insulin sensitivity (23–25). Additionally, a large retrospective cohort study conducted at Xiamen University, which included 13,745 intrauterine insemination cycles, found that women with low body weight had lower cumulative pregnancy and live birth rates compared with normal-weight women. The study concluded that low body weight may impair endometrial receptivity (26). Souter et al. also reported a positive correlation between BMI and endometrial thickness, suggesting that lower BMI may be a detrimental factor for endometrial receptivity (27). Furthermore, an animal study demonstrated that decreased oocyte quality in a lean mouse model of PCOS was associated with impaired mitochondrial ultrastructure and function (28), which may offer insights for future mechanistic investigations. In summary, considering the BMI inflection point identified in our study, increasing preconception BMI—particularly through muscle-building interventions—may improve pregnancy outcomes in PCOS patients with BMI < 22 kg/m², especially in younger individuals.

According to the Chinese Obesity Working Group classification system (11), patients with PCOS were categorized into underweight, normal weight, overweight, and obese groups. Although live birth rates in the overweight and obese groups were not significantly different from those in the normal weight group, a decreasing trend was observed, consistent with previous research findings (5–7, 12, 17). This supports the concept that obese patients with PCOS tend to have lower live birth rates compared with those of normal weight. Previous studies have suggested that individuals with obesity and PCOS are more likely to exhibit insulin resistance, which is an independent risk factor for early miscarriage (10, 29). Moreover, obesity is widely recognized for its adverse effects on both the quantity and quality of oocytes, as well as for reducing endometrial receptivity—ultimately impairing embryo implantation and lowering clinical pregnancy rates (30). Although our study did not demonstrate a statistically significant reduction in live birth rates among individuals with high BMI, the presence of obesity-related metabolic disturbances still warrants BMI optimization prior to conception. This may help reduce the risk of maternal and neonatal complications, as well as adverse effects on offspring (31, 32).

This study has several strengths. First, it exclusively included patients with PCOS who underwent fresh embryo transfers, thereby reducing the influence of certain confounding variables. Second, the association between BMI and live birth rate was further stratified by age, allowing for subgroup analysis. However, several limitations should be acknowledged. First, the retrospective study design limits causal inference, even though the temporal sequence between BMI and pregnancy outcomes was established. Second, the sample size was relatively small, with only 6.8% of participants having a BMI < 18.5 kg/m² and 9.5% having a BMI ≥ 28 kg/m², which may have influenced the robustness of subgroup analyses. Nevertheless, the median BMI was 22.5 kg/m², and 260 patients (40%) had a BMI < 22 kg/m², which provided adequate power for evaluating associations in this subgroup. Third, body composition analysis was not performed for all participants, resulting in a lack of data on fat percentage and muscle mass. Moreover, BMI does not distinguish between subcutaneous and visceral adipose tissue. Future studies should incorporate direct assessments of body composition. Fourth, this study only included fresh embryo transfer cycles. A parallel investigation that includes frozen embryo transfer cycles is currently underway, which will improve the generalizability of the findings.

## Conclusion

This study was the first to propose a lower threshold for BMI management (BMI = 22 kg/m²) in Chinese patients with PCOS, highlighting the need for clinicians to consider weight-related risks at both ends of the BMI spectrum. Specifically: (1) Young PCOS patients with a BMI < 22 kg/m² should undergo nutritional assessment and receive individualized weight gain recommendations; (2) For individuals with obesity aged over 32 years, BMI should be optimized prior to conception. In summary, our study demonstrated for the first time that low BMI in PCOS patients is associated with adverse pregnancy outcomes. These findings underscore the importance of recognizing low BMI as a risk factor in reproductive planning and offer a new clinical perspective for individualized weight management and assisted reproductive strategies in patients with PCOS.

## Data Availability

The data analyzed in this study is subject to the following licenses/restrictions: Some data involves patients privacy. Requests to access these datasets should be directed to Fenghua Liu, liushine2006@163.com.
